# Loss of CASZ1 tumor suppressor linked to oncogenic subversion of neuroblastoma core regulatory circuitry

**DOI:** 10.1038/s41419-022-05314-6

**Published:** 2022-10-15

**Authors:** Zhihui Liu, Xiyuan Zhang, Man Xu, Haiyan Lei, Jack F. Shern, Carol J. Thiele

**Affiliations:** Pediatric Oncology Branch, National Cancer Institute, Bethesda, MD USA

**Keywords:** Paediatric cancer, Diseases

## Abstract

The neural crest lineage regulatory transcription factors (TFs) form a core regulatory circuitry (CRC) in neuroblastoma (NB) to specify a noradrenergic tumor phenotype. Oncogenic subversion of CRC TFs is well documented, but the role of loss of tumor suppressors plays remains unclear. Zinc-finger TF CASZ1 is a chromosome 1p36 (chr1p36) tumor suppressor. Single-cell RNA sequencing data analyses indicate that CASZ1 is highly expressed in developing chromaffin cells coincident with an expression of NB CRC TFs. In NB tumor cells, the CASZ1 tumor suppressor is silenced while CRC components are highly expressed. We find the NB CRC component HAND2 directly represses CASZ1 expression. ChIP-seq and transcriptomic analyses reveal that restoration of CASZ1 upregulates noradrenergic neuronal genes and represses expression of CRC components by remodeling enhancer activity. Our study identifies that the restored CASZ1 forms a negative feedback regulatory circuit with the established NB CRC to induce noradrenergic neuronal differentiation of NB.

## Introduction

Neuroblastoma (NB) is the most common extracranial pediatric solid tumor and is derived from the neural crest cells, which results in tumors in the adrenal glands and/or sympathetic ganglia [1–3]. Despite extensive multi-modal treatment, the long-term survival of high-risk NB is still under 40% [3, 4]. In embryonal tumors such as NB, oncogenic events are thought to involve the hijacking of developmental processes that favor self-renewal over differentiation [1]. Tumors from low-risk NB patients present with a range of differentiated cell types are rich in stroma, while those from high-risk NB patients are monotonous sheets of undifferentiated neuroblasts with few stromal cells. In addition to histologic differences, NB tumors are known to have extensive intra-tumoral heterogeneity with studies focused on two major subtypes named noradrenergic or mesenchymal NB, based on their distinct mRNA profiles and core transcriptional regulatory circuits (CRC) [5, 6]. Most cultured NB cell lines and patient-derived NB tumors are found to have a noradrenergic but not a mesenchymal character based on both the bulk RNA sequencing (RNA-seq) and the single-cell RNA-seq data analysis [5–9]. The CRC components of noradrenergic NB include transcription factors (TFs) ISL1, HAND2, GATA3, PHOX2B, TBX2, and ASCL1, and these TFs form an interconnected autoregulatory feed-forward loop that enforces a malignant noradrenergic phenotype of NB [5, 6, 10–12]. This is consistent with the concept that cell-fate decisions and cell identities are determined by a limited number of TFs that regulate each other and their downstream target genes [13, 14]. However, how the CRCs switch during tumorigenesis, and if a tumor cell silences natural regulatory mechanisms that might repress a tumor cell CRC through depletion of normal cell core TFs during cell identity-switch is unknown.

The *CASZ1* gene encodes a zinc-finger TF that orchestrates cell-fate specification, commitment, and differentiation in neuroblasts, retinal progenitors, T helper cells, cardiac progenitors, and cardiomyocytes during normal development [15–23]. Human *CASZ1* localizes to chromosome band 1p36 (Chr1p36), and loss of heterozygosity of this region (1pLOH) is implicated in many types of cancers including NB [24]. Low expression of *CASZ1* is detected in poorly differentiated NB cancer cells [25–29] and *CASZ1* is negatively regulated by PRC2 complex [25–30]. Human *CASZ1* gene encodes two isoforms: the CASZ1b isoform is the more evolutionarily conserved isoform with 1166 amino acids (AA) and 5 zinc fingers, while CASZ1a has 1,759 AA that contains the exact sequence of CASZ1b plus 6 more zinc fingers [31]. Both isoforms function similarly at regulating gene transcription and suppressing neuroblastoma growth [25, 26, 32]. Our recent work demonstrates that CASZ1b isoform induces skeletal myogenesis and embryonic rhabdomyosarcoma differentiation by forming a feed-forward regulatory circuit with myogenic differentiation TFs, MYOD, and MYOG [33]. However, how the tumor suppressor CASZ1b regulates gene transcription at an epigenetic level to suppress NB tumor growth has not been investigated. Moreover, it is not known whether tumor suppressor genes impact the NB CRC and if they do what are the regulatory mechanisms.

In this study, we performed loss and gain of function studies of *CASZ1* in neuroblastoma cell lines paired with detailed epigenetic and transcriptomic characterization. For the gain of *CASZ1* function study, we overexpressed CASZ1b isoform since it is the more evolutionarily conserved and has a similar biological function as the CASZ1a isoform in NB. We found that CASZ1b directly upregulates noradrenergic differentiation genes and represses mesenchymal genes and regulators of the NB cell cycle. Importantly, we found that the CRC TFs in NB repress *CASZ1* expression and restoration of CASZ1b suppresses expression of the noradrenergic CRC TFs. Thus, CASZ1 forms a negative feedback loop with CRC TFs and its loss of expression shapes the tumorigenic NB cell identity. These studies identify for the first time how loss of a tumor suppressor with TF activity is linked to the oncogenic subversion of a lineage specifying CRC.

## Results

### CASZ1 is essential for neuronal differentiation of NB

**Fig. 1:**
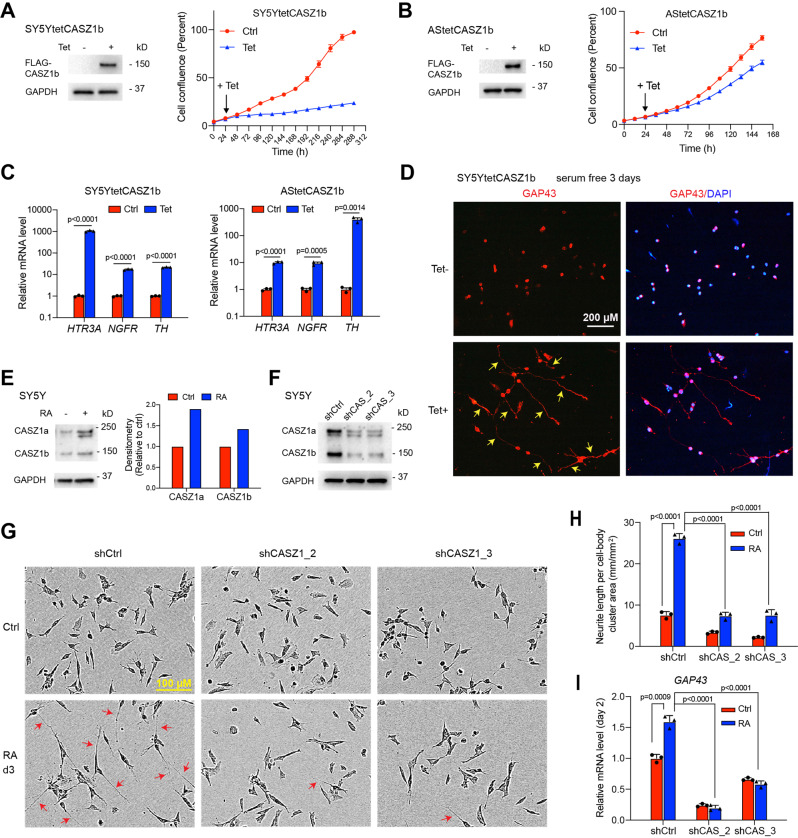
CASZ1b is essential for neuronal differentiation. **A** Western blot shows induction of CASZ1b expression in CASZ1b stably transfected SY5Y cells after tetracycline (Tet) treatment for 24 h (left panel); CASZ1b represses SY5Y cell proliferation is observed based on IncuCyte cell confluence assay. **B** Western blot shows induction of CASZ1b expression in CASZ1b stably transfected AS cells after Tet treatment for 24 h (left panel); CASZ1b represses AS cell proliferation is observed based on IncuCyte cell confluence assay (right panel). **C** Realtime PCR results show that the restoration of CASZ1b upregulates mRNA levels of neuronal genes in both SY5Y cells (48 h Tet treatment) and AS cells (72 h Tet treatment). **D** CASZ1b induces neurite extension (yellow arrow) in SY5Y cells shown by GAP43 staining. **E** Western blot shows that CASZ1 is upregulated when SY5Y cells are treated with retinoic acid for 3 days. The densitometry graph on the right is generated by normalizing GAPDH signal and the signal in Ctrl is set as 1. **F** Western blot shows that the knockdown of CASZ1 decreases expression of both CASZ1a and CASZ1b isoforms. **G** The knockdown of CASZ1 attenuates RA-induced neurite extension (red arrow) shown by the phase-contrast imaging. **H** The silencing of CASZ1 results in a significant decrease in RA-induced neurite length as evaluated by using the IncuCyte neurite-length assay. **I** Realtime PCR results show that the siRNA knockdown of CASZ1 for 2 days attenuates RA induced increases in GAP43 mRNA levels. Data represent mean ± SEM, *n* = 3 biological replicates. Two-sided Student’s *t* test was used to calculate statistical difference.

SH-SY5Y is a noradrenergic type of NB cell line and our previous study showed that the restoration of CASZ1b in SY5Y cells results in decreased cell proliferation [26]. The SK-N-AS (AS) NB cell line has a mixed noradrenergic and mesenchymal phenotype [5]. Using tetracycline (Tet) inducible CASZ1b overexpressing SY5Y (SY5YtetCASZ1b) and AS (AStetCASZ1b) cell lines, we found that the induction of CASZ1b suppressed both SY5Y and AS cell growth (Fig. 1A, B). Realtime PCR results showed that the induction of CASZ1b in both SY5Y and AS cells significantly upregulated the expression of neuronal genes including serotonin 3A receptor (HTR3A) [34], nerve growth factor receptor (NGFR) [35], and tyrosine hydroxylase (TH) [36] (Fig. 1C). Induction of CASZ1b did not induce morphologic differentiation in AS cells, but in SY5Y cells induction of CASZ1b resulted in neurite extension as detected by anti-growth associated protein 43 (GAP43) antibody staining (Fig. 1D). We have previously shown that retinoic acid (RA) treatment of SY5Y cells results in an upregulation of both *CASZ1a* and *CASZ1b* at mRNA levels [26]. Here we found that the RA treatment of SY5Y cells for 3 days resulted in an upregulation of both CASZ1a and CASZ1b at protein levels shown by western blot analysis (Fig. 1E). To understand the role of *CASZ1* in RA induced cell differentiation, we silenced *CASZ1* using two different short hairpin RNAs (shRNAs) in SY5Y cells (Fig. 1F). While a significant number of neurites were observed in RA treated, shRNA control vector (SY5YshCtrl) transduced cells, few neurites (Fig. 1G) and reduced neurite length per cell-body (Fig. 1H) were observed in RA treated, CASZ1 shRNA (SY5YshCASZ1) transduced cells (Fig. 1G, H). RA treatment resulted in an increase in the neuronal marker GAP43 mRNA levels in shCtrl vector transduced cells but not in shCASZ1 transduced cells (Fig. 1I). These results indicate that CASZ1 is essential for NB cell differentiation.

### CASZ1 regulates sympathoadrenal lineage genes

**Fig. 2:**
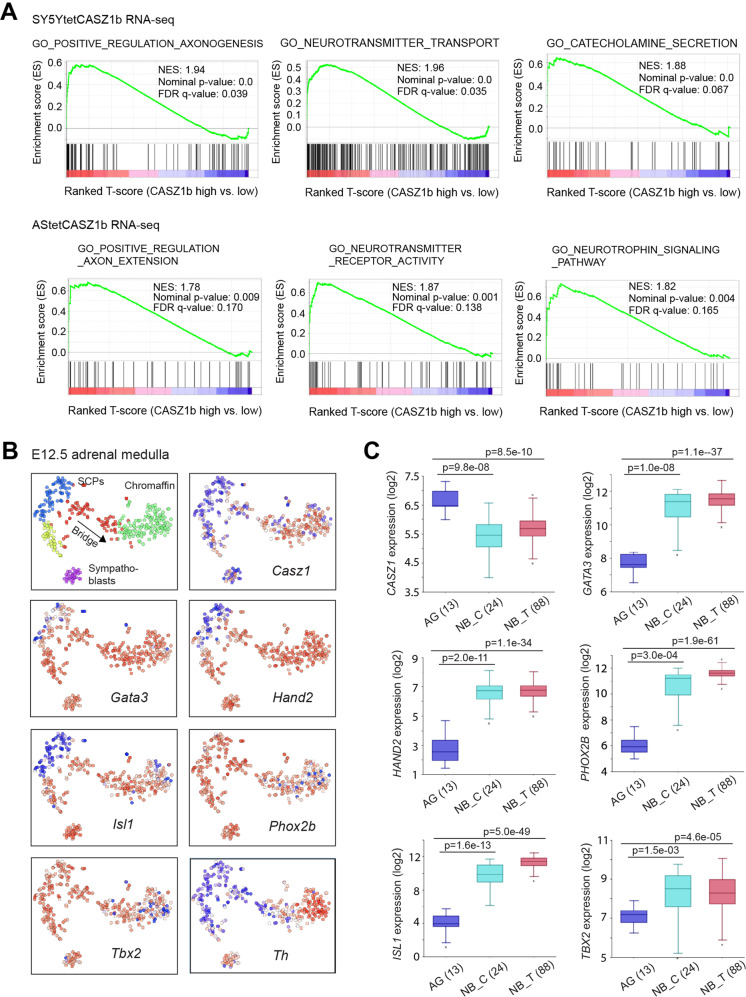
CASZ1 regulates sympathoadrenal lineage genes. **A** GSEA shows a positive enrichment of axonogenesis, neurotransmitter transport, and catecholamine secretion genes upon induction of CASZ1b in SY5Y cells (top panel). GSEA shows a positive enrichment of axonogenesis, neurotransmitter transport and neurotrophin signaling genes when CASZ1b is overexpressed in AS cells. **B** The single-cell mRNA expression pattern of *Casz1*, CRC components, and sympathoadrenal lineage-determination and mark genes in E12.5 mouse embryonic adrenal medulla were analyzed using the Harvard interactive interface tools (http://pklab.med.harvard.edu/cgi-bin/R/rook/nc.SS2_16_250-2/index.html; scRNAseq results are available at Gene Expression Omnibus, GSE99933). Note: left-upper corner image: SCPs, schwann precursor cells (blue); SCPs transition to an intermediate cell population called Bridge cells (red) as they transition to chromaffin cells (green) or Bridge cells (yellow) as they transition to Sympathoblasts (purple). The relative expression magnitude: blue low, white intermediate, and red high. **C** The mRNA levels of *CASZ1* and CRC components in human normal adrenal gland (AG), neuroblastoma cell lines (NB_C) and neuroblastoma tumors (NB_T, Versteeg cohort) are analyzed using the R2:Genomics Analysis and Visualization platforms (gserver1.amc.nl/cgi-bin/r2/main.cgi). Data are presented as box and whisker plots with middle lines indicating medians and whiskers representing the 25th and 75th percentiles. The graph is generated using databases in the R2 platform.

To interrogate the transcriptional consequences of CASZ1 expression in NB cells, we performed RNA sequencing (RNA-seq) analysis of SY5Y cells and AS cells with or without restoration of CASZ1b and identified transcriptome regulated by CASZ1b in these two NB cell lines (Supplementary Table 1). Gene set enrichment assay (GSEA) of RNA-seq data showed that restoration of CASZ1b in SY5Y cells resulted in a positive enrichment of genes that positively regulate axonogenesis (Fig. 2A, top panel). Moreover, CASZ1b over-expression led to a significantly positive enrichment of genes regulating neurotransmitter transport and catecholamine secretion, which are expressed in the differentiating and differentiated noradrenergic cells. Although the transcriptome regulated by CASZ1b in AS cells was not completely the same as in SY5Ycells, GSEA showed that restoration of CASZ1b in AS cells also results in a positive enrichment of neuronal genes such as genes positively regulate axon extension, neurotransmitter receptor activity and neurotrophin signaling (Fig. 2A, bottom panel). Our results indicate that CASZ1b induces a sympathoadrenal differentiation program of NB cells.

Primary NB commonly arises in neural crest cells of the sympathoadrenal lineage. A recent study of early adrenal medulla development in mouse embryos using single-cell RNA sequencing (scRNA-seq) [37] provides an opportunity to investigate the expression pattern of *Casz1* in the normal developing murine sympathoadrenal cells at the single-cell level. By analyzing scRNA-seq data of E12.5 murine embryos, we found that *Casz1* is expressed at relatively low levels in Schwann cell precursors (SCPs) and sympathoblasts yet *Casz1* levels rise in transitional stage cells (bridge) and reach their highest relative levels in the developing and differentiated chromaffin cells (Fig. 2B). Adrenal medullary cells clustered based on gene expression (Supplementary Fig. 1A, top panel) were enriched in genes co-expressed with *Casz1* (Supplementary Fig. 1A, bottom panel), which are similar to those clusters of cells that are enriched in neuron projection and neurotransmitter genes (Supplementary Fig. 1A, top panel). GO pathway enrichment assay of genes co-expressed with Casz1 showed a positive enrichment of synapse associated genes (Supplementary Fig. 1B). The noradrenergic NB CRC components including *Phox2b, Gata3, Hand2, Isl1,* and *Tbx2* are known TFs that play important roles in regulating sympathoadrenal lineage development. We found these genes and other neural crest regulators including *Gata2, hand1*, and *phox2a* were all expressed in sympathoblasts, transitional stage cells and developing chromaffin cells, with variable mRNA levels in SCPs (Fig. 2B, Supplementary Fig. 1C). These observations suggest that *Casz1* is involved in normal adrenal medullary development and cooperates with those CRC components to regulate chromaffin cell differentiation.

We next compared the expression of *CASZ1* and CRC components in human adrenal gland (AG), neuroblastoma cell lines and neuroblastoma tumors (Versteeg cohort) [38] by analyzing the R2 database (https://hgserver1.amc.nl/cgi-bin/r2/main.cgi). We found that the relative mRNA levels of CASZ1b were lower in NB cell lines and NB tumors than in normal adrenal gland derived cells. However, the relative mRNA levels of the CRC components such as GATA3 and HAND2 were significantly higher in NB tumors than in adrenal gland derived cells (Fig. 2C). This is consistent with the function of CASZ1b as a tumor suppressor in NB [26], while CRC components are genes essential to maintain the oncogenic noradrenergic phenotype of NB cells [10]. Our results indicate a reverse correlation between CASZ1 and CRC TFs in NB cells.

### CASZ1 is transcriptionally regulated by CRC components

**Fig. 3:**
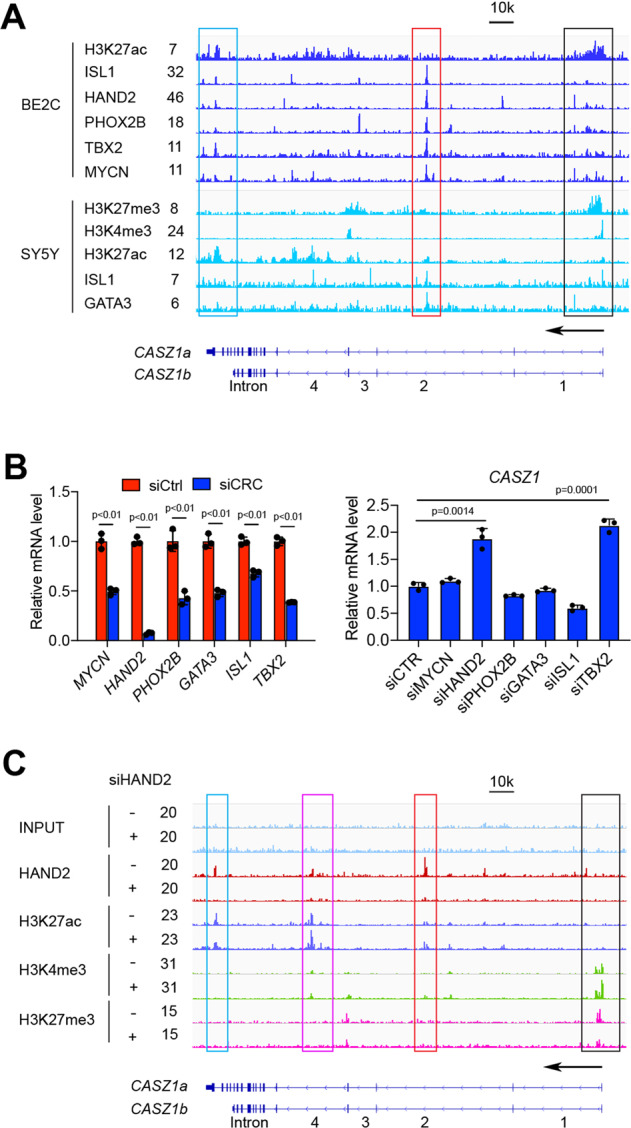
CASZ1 is regulated by noradrenergic NB CRC components. **A** Results of analysis of publicly available ChIP-seq data of the CRC components show that the CRC components bind to the *CASZ1* gene locus, with a relatively stronger signal of CRC components within the 2nd intron (red box) but a lower signal of H3K27ac. Bivalent mark of H3K27me3 and H3K4me3 is observed at the *CASZ1* gene promoter in SY5Y cells (black box). **B** Realtime PCR shows that the siRNA knockdown of MYCN and CRC compoents (left panel), and the loss of *HAND2* or *TBX2* results in a decrease of *CASZ1* mRNA levels (right panel). **C** ChIP-seq results show that decreasing expression of *HAND2* in IMR32 cells results in a decrease of HAND2 signal within *CASZ1* gene locus, which is accompanied by an increase of H3K27ac signal within the 2nd and 3rd intron (red and pink boxes), as well as an increase of H3K4me3 signal and a decrease of H3K27me3 signal within the *CASZ1* promoter.

To investigate whether the high expression of NB CRC components impacts *CASZ1* expression, we first analyzed publicly available CRC TFs ChIP-seq data of noradrenergic NB cell lines BE(2)C and SY5Y [10, 39]. We found that all the CRC TFs bound to the *CASZ1* gene locus with multiple peaks shown by the signal tracks (Fig. 3A). The binding sites of these TFs within *CASZ1* gene locus included the promoter (Fig. 3A, black box), and the 3′ terminus (Fig. 3A, cyan box) with the strongest signal within the 2nd intron (Fig. 3A, red box). Interestingly, the active enhancer mark H3K27ac signal within the 2nd intron (Fig. 3A, red box) was lower compared to the other regions in BE2C and SY5Y cells, which suggests that these TFs might play a repressive role in regulating CASZ1 gene expression. Knockdown the CRC genes *HAND2* or *TBX2* but no other CRC components in the noradrenergic NB cell line IMR32 (*MYCN*-amplified), led to a significant increase in *CASZ1* mRNA expression (Fig. 3B). Upon selectively silencing of *HAND2* expression, followed by ChIP-seq using anti-HAND2, H3K27ac, H3K4me3 and H3K27me3 antibodies [40], we found a decrease in HAND2 binding within the CASZ1 gene locus compared to controls, and an increase in H3K27ac signal within the 2nd and 4th intron (Fig. 3C). Loss of HAND2 resulted in an increase of H3K4me3 signal and a decrease of H3K27me3 signal at the TSS region of *CASZ1* gene, suggesting that the changes of H3K4me3 and H3K27me3 signal might be mediated through PRC2 complex. *CASZ1* has been reported to be repressed by PRC2 complex and a bivalent mark of H3Kme3 and H3K27me3 has been found at the transcription start site (TSS) of *CASZ1* gene [29].

### Genome-wide binding of CASZ1 in NB cells

**Fig. 4:**
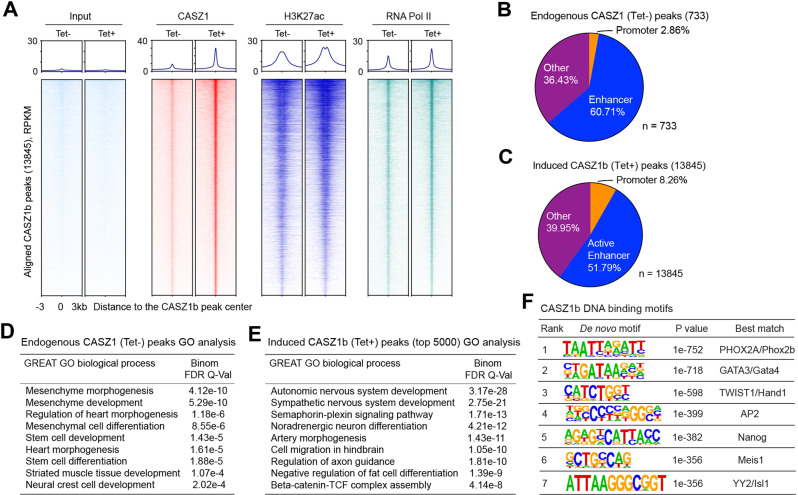
Genome-wide mapping of CASZ1b binding sites. **A** Heatmap shows the ranked CASZ1b binding peaks (13,845) and the aligned peaks of H3K27ac, RNA Pol II and input control at CASZ1b peak center before (−) and after (+) Tet treatment of SY5YtetCASZ1b cells. RPKM, reads per kilobase per million. **B** Endogenous CASZ1 peak distribution shows that CASZ1 mainly binds to active enhancers. **C** Induced CASZ1b peak distribution analysis shows that CASZ1b mainly binds to active enhancers. **D** GREAT GO biological process analysis indicates endogenous CASZ1 binding sites are associated with mesenchyme, heart and neural crest development. **E** GREAT GO biological process analysis shows that the CASZ1b binding sites are associated with sympathetic nervous system development and noradrenergic neuron differentiation. **F** Homer de novo motif scan of CASZ1b binding sites shows the enrichment of neural crest development associated TFs and NB noradrenergic CRC TFs binding motifs. RPKM: reads per kilobase per million mapped reads.

To gain insights into how the restoration of cellular levels of CASZ1 affects the epigenome, we performed ChIP-seq experiments using anti-CASZ1, H3K27ac, and RNA Pol II antibodies in SY5YtetCASZ1 cells treated with or without Tet for 2 days. We identified 733 CASZ1 binding sites (narrow peak calling, *p* < 10^−7^) that associated with 614 genes (Supplementary Table 2) in the cells without Tet treatment (Tet−). This represents the basal, endogenous CASZ1 binding sites. Upon induction of CASZ1b (Tet+), we identified 13845 Tet-induced CASZ1b peaks that associate with 7525 genes (Supplementary Table 2). These sites had a dramatic increase in the CASZ1b peak centered signal compared to control cells as shown in the ChIP-seq heatmap (Fig. 4A). We further investigated changes in the chromatin landscape after CASZ1b induction to understand in more mechanistic detail how CASZ1 regulates gene transcription. ChIP-seq heatmaps showed that Tet-induced CASZ1b peak centers overlapped with H3K27ac and RNA Pol II binding sites (Fig. 4A). After the restoration of CASZ1b in SY5Y cells, all CASZ1b-bound peaks showed a measurable increase of H3K27ac and RNA Pol II signals (Fig. 4A). Endogenous CASZ1 peak distribution analysis showed that 2.86% of CASZ1 binding sites were at promoter regions (defined by ±1 kb of transcription start site, TSS). Overlaying the promoter-distal H3K27ac peaks, which mark active enhancers, with the endogenous CASZ1 peaks, we found that 60.71% CASZ1 binding sites were within active enhancers (Fig. 4B). Tet-induced CASZ1b peak distribution analysis showed that 8% of CASZ1b binding sites were within promoter regions while 51.8% CASZ1b binding sites were within active enhancers (Fig. 4C).

To characterize the CASZ1 binding sites associated genes, Genomic Regions Enrichment of Annotations Tool (GREAT) [41] was used for gene ontology (GO) analysis of both the endogenous CASZ1 peaks and Tet-induced CASZ1b peaks. The results showed that at baseline the endogenous CASZ1 bound peak-associated genes were significantly enriched in mesenchyme development and heart morphogenesis (Fig. 4D). HOMER *de novo* and known motif scan of the endogenous CASZ1 binding sites identified an enrichment of GATA family TFs, ASCL1, PHOX2 A binding motifs (Supplementary Fig. 2A, B). GREAT GO analysis showed that when the levels of CASZ1 expression increased, genes associated with Tet-induced CASZ1b peaks were enriched in sympathetic nervous system development and noradrenergic neuron differentiation (Fig. 4E). Like endogenous CASZ1, HOMER *de novo* and known motif scan of the Tet-induced CASZ1b binding sites identified an enrichment of GATA family TFs, PHOX2A, PHOX2B, HAND1 binding motifs (Fig. 4F, Supplementary Fig. 2C). These TFs are known neural crest lineage specifiers [42, 43], which suggests a role for CASZ1 in regulating normal neural crest development.

### CASZ1b directly upregulates neuronal genes

**Fig. 5:**
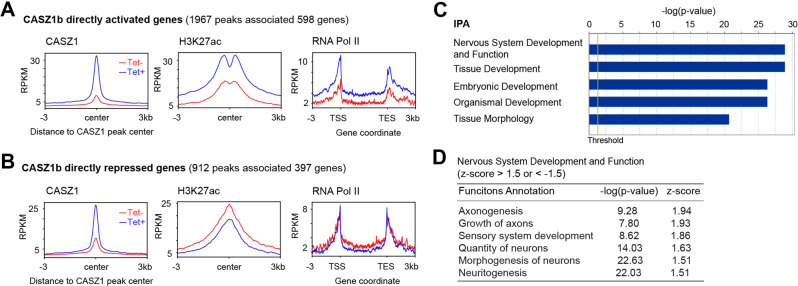
CASZ1b directly activates neuronal differentiation genes. **A** Composite plots of the 598 upregulated genes before (Tet-) after (Tet+) induction of CASZ1b in SY5Y cells, which show increased CASZ1b signals (left panel) and H3K27ac signals (middle panel) at the CASZ1b peak center, as well as increased signals of RNA Pol II on the gene body (right panel). **B** Composite plots of the 397 genes directly downregulated by CASZ1, which show increased CASZ1b signals (left panel) and decreased signals of H3K27ac (middle panel) at the CASZ1b peak center, as well as decreased signals of RNA Pol II on the gene body (right panel). **C** Ingenuity pathway analysis (IPA) shows that CASZ1b directly regulated genes are involved in nervous system and tissue development. **D** IPA shows that CASZ1b directly regulated genes are enriched in the positive regulation of neuronal differentiation and axonogenesis.

To understand the regulatory consequences of CASZ1b binding, we focused on genes that were genomically bound and transcriptionally regulated by CASZ1b. To accomplish this, RNA-seq data and CASZ1b ChIP-seq data from SY5YtetCASZ1b cells with and without induced CASZ1b expression (2-day treatment with Tet) were merged. In this analysis, 1967 sites associated with 598 genes were bound and transcriptionally upregulated by CASZ1b (Fig. 5A, Supplementary Table 3), while 912 sites associated with 397 genes were bound and transcriptionally downregulated by CASZ1b (Fig. 5B, Supplementary Table 3). We found that after the restoration of CASZ1b in NB cells, there was an increase of RNA Pol II signal within the gene body of CASZ1b upregulated genes and a decrease of RNA Pol II signal within the gene body of CASZ1b downregulated genes (Fig. 5A, B). For upregulated genes, we found that there was an increase of the active enhancer mark H3K27ac signal at Tet-induced CASZ1b peak center (Fig. 5A), indicating that CASZ1b increased enhancer activity to upregulate gene transcription. For genes directly downregulated by CASZ1b, there was a corresponding decrease of H3K27ac signal at Tet-induced CASZ1b peak centers (Fig. 5B). Interestingly, unlike traditional H3K27ac peaks observed in CASZ1b upregulated genes (Fig. 5A), no bimodal H3K27ac peaks (“valley” pattern) were observed at the Tet-induced CASZ1b peak center for CASZ1b downregulated genes (Fig. 5B). Ingenuity pathway analysis (IPA) revealed genes directly regulated by CASZ1b (both upregulated and downregulated) were involved in nervous system development and developmental pathways (Fig. 5C). By focusing on the genes involved in the nervous system development, IPA showed that neuronal differentiation genes were positively enriched as indicated by the positive z-score (Fig. 5D), consistent with the observation of induction of neurite extensions in SY5Y cells after the restoration of CASZ1b (Fig. 1D).

### CASZ1b directly represses mesenchyme development genes, CRC components and activates neuronal genes through remodeling typical enhancers (TEs) and super-enhancers (SEs)

**Fig. 6:**
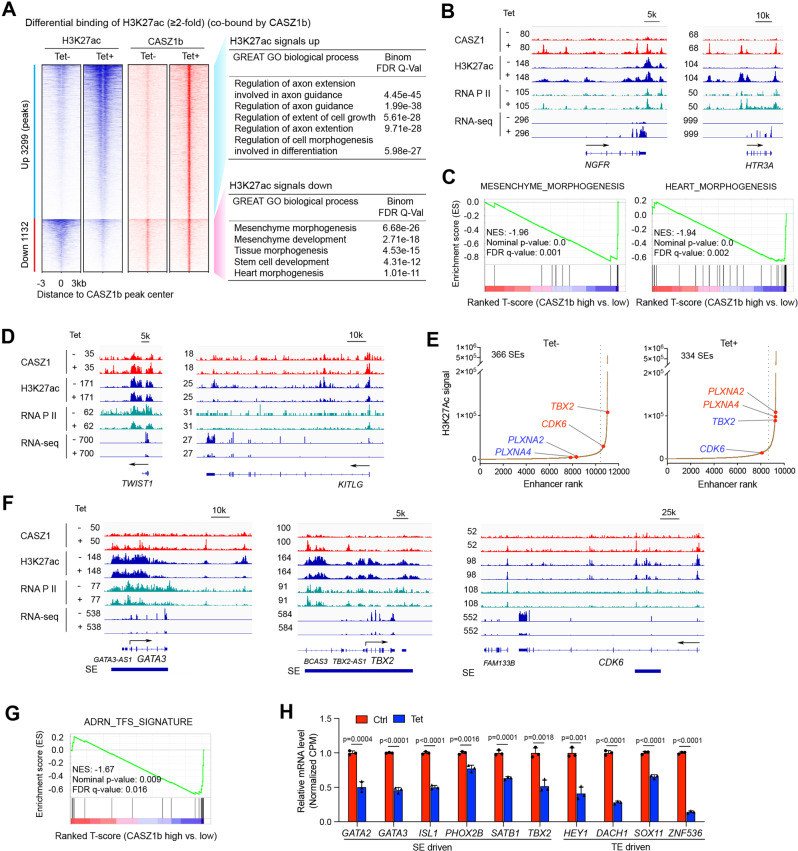
CASZ1b represses NB CRC and mesenchymal signature genes and activates neuronal genes through affecting enhancer activity. **A** Restoration of CASZ1b in SY5Y cells results an increase of H3K27ac signals (heatmap on the left) on genes that are associated with axonogenesis (GO analysis on the right) and a decrease of H3K27ac signals on genes (heatmap on the left panel) that are associated with mesenchyme development (GO analysis, right panel). **B** Signal tracks show the increase of CASZ1b, H3K27ac and RNA Pol II signals and RNA-seq signals on the neuronal genes *NGFR* and *HTR3A* after induction of CASZ1b (Tet+ vs. Tet−). **C** GSEA shows a negative enrichment of mesenchyme morphogenesis and heart morphogenesis genes upon induction of CASZ1b in SY5Y cells. **D** Signal tracks show the increase of CASZ1b signals, and decrease of H3K27ac, RNA Pol II and RNA-seq signals on the mesenchyme development regulators *TWIST1* and *KITLG* after induction of CASZ1b (Tet+ vs. Tet−). **E** Restoration of CASZ1b in SY5Y cells re-organizes SEs. The histograms show an increase of H3K27ac signal on neuronal genes *PLXNA2* and *PLXNA4* and a decrease of H3K27ac signals on CRC component *TBX2*, as well as cell cycle progression regulator *CDK6*. **F** Signal tracks show that the restoration of CASZ1b in SY5Y cells results in a decrease of SE signals (H3K27ac), as well as RNA Pol II signals and RNA-seq reads on *GATA3* and *CDK6*; **G** GSEA shows that the restoration of CASZ1b in SY5Y cells results in a significant negative enrichment of noradrenergic NB TFs coding genes. **H** The bar graph shows the negative regulation of noradrenergic NB TFs coding genes that either driven by SEs or typical enhancers (TEs) in SY5Y cells after restoration of CASZ1b based on the normalized RNA-seq reads (CPM: counts per million). Data represent mean ± SEM, *n* = 3 biological replicates. Two-sided Student’s *t* test was used to calculate statistical difference.

Enhancers play a central role in driving cell-type-specific gene expression through activating transcription of their target genes that may be several to hundred kilobases or even megabases away [44]. To investigate the pathways that are regulated by CASZ1b-bound enhancers, we focused on those peaks co-bound by both CASZ1b and H3K27ac with changes in their H3K27ac signals (>2-fold) after CASZ1b restoration. We found that 3299 H3K27ac peaks associated with 2554 genes had at least a 2-fold increased signal upon CASZ1b induction (Tet+) compared to control cells (Tet−). In contrast 1132 H3K27ac peaks associated with 1293 genes had at least a 2-fold decreased signal (Fig. 6a, Supplementary Table 4). GREAT GO analysis of the H3K27ac peaks with increased signals after CASZ1b induction showed enrichment of genes involved in nervous system development (Fig. 6A), which is consistent with RNA-seq results showing CASZ1b upregulates neuronal genes (Fig. 2A). Consistently, signal tracks showed that the induction of CASZ1b in SY5Y cells resulted in *de novo* CASZ1b binding, as well as an increase of H3K27ac, RNA Pol II ChIP-seq signals and RNA-seq signal on the neuronal gene *NGFR* and *HTR3A* (Fig. 6b). GREAT GO analysis of the H3K27ac peaks with decreased signals showed enrichment of genes involved in mesenchyme morphogenesis and development (Fig. 6A). Noradrenergic NB cells like SY5Y highly express a noradrenergic gene signature but not a mesenchymal gene signature [6], leading us to hypothesize that CASZ1b binds to mesenchymal gene loci to repress their expression. Indeed, GSEA showed that the restoration of CASZ1b in SY5Y cells resulted in a negative enrichment of mesenchyme morphogenesis and development genes, heart morphogenesis genes, and striated muscle tissue development genes (Fig. 6C, Supplementary Fig. 3A), consistent with an association of CASZ1 with a repressive role on genes regulating mesenchyme development. Additionally, signal tracks showed that the induction of CASZ1b in SY5Y cells resulted in a decrease of H3K27ac, RNA Pol II ChIP-seq signals and RNA-seq signals on genes regulating mesenchyme development such as *TWIST1* and *KITLG* (Fig. 6D).

**Fig. 7:**
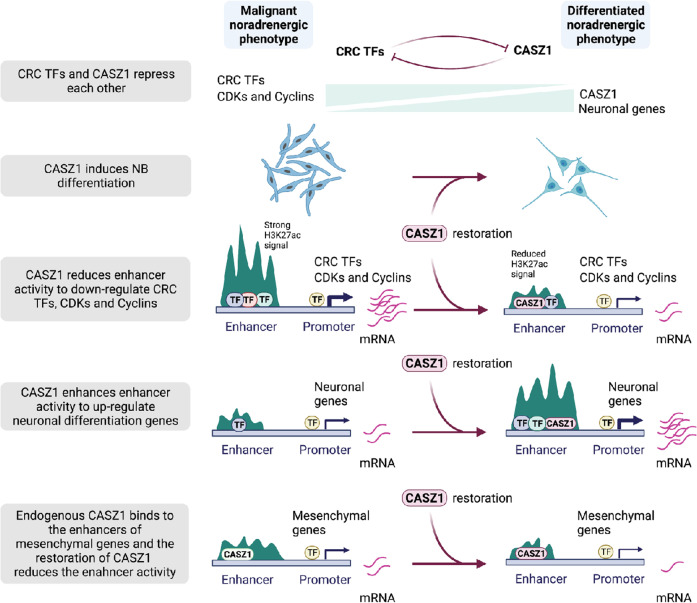
Schematic diagram of CASZ1 action. Restoration of CASZ1 directly represses CRC TFs, suppresses cell cycle regulators, mesenchymal genes and activates neural differentiation genes through remodeling enhancers.

Cell-fate and cell identity are determined by super-enhancers (SEs) that are marked by extensive stretches of H3K27ac and these SEs are frequently dysregulated in cancer [45]. Our study identified 366 SEs in the control cells (Tet-) and 344 SEs in CASZ1b restored SY5Y cells (Tet+) (Fig. 6E, Supplementary Table 5). CASZ1b peaks were found in over 90% SEs in CASZ1b restored cells (Supplementary Fig. 3b). CASZ1b re-established SEs shown by both the gain and loss of SEs upon CASZ1b induction in SY5Y cells (Fig. 6E, Supplementary Fig. 3C, Supplementary Table 5). Ingenuity Pathway Analysis indicated that both gained and lost SEs associated genes were enriched in cellular development and growth regulation (Supplementary Fig. 3D). However, when focused on these categories in detail by looking into the function annotations, we found that the lost SEs associated genes after the restoration of CASZ1b are enriched in regulating “cell proliferation of tumor cell lines” (Supplementary Fig. 3D, top panel), which includes cell cycle regulators CCND1 and CDK6 that are known growth regulators in NB [46, 47]. The gained SEs associated genes are enriched in “development of neurons” and “growth of neurites” (Supplementary Fig. 3D, middle panel), while the common SEs associated genes are enriched in “cell proliferation of tumor cell lines” and “synthesis of norepinephrine” (Supplementary Fig. 3D, bottom panel). By focusing on the SEs that drive the CRC components and neural crest lineage specifiers, we observed that the restoration of CASZ1 resulted in a decrease of SEs signals (H3K27ac signals) on a subset of these TFs, including GATA2, GATA3, ISL1 and TBX2 (Fig. 6F, Supplementary Fig. 3E). CASZ1 also decreased the H3K27ac signals within the *CDK6* gene locus (Fig. 6F). *CDK6* regulates the progression of cell cycle and is a potential therapeutic target in NB [47]. In a previous study we showed that the restoration of CASZ1b in NB cells led to a decrease in the protein expression of cell cycle regulators such as CDK1, CDK6 and Cyclin D1 (CCND1), but the mechanism was unknown [27]. Here we found that CASZ1b directly repressed the expression of these cell cycle regulators by binding to the genomic loci of these genes, with the increased CASZ1b binding accompanied by decreased H3K27ac signals (Fig. 6F, Supplementary Fig. 3F). Similarly, we found that CASZ1b directly repressed MYC gene expression by binding to the MYC gene locus (Supplementary Fig. 3F). Moreover, the restoration of CASZ1 resulted in a gain of SEs on neuronal genes such as *PLXNA2* and *PLXNA4* (Supplementary Fig. 3G). Finally, we focused on changes in the expression of TFs that were enriched in noradrenergic NB cells [6, 10, 11] upon CASZ1b restoration. GSEA demonstrated that these noradrenergic lineage TFs were negatively enriched when CASZ1b is restored in SY5Y cells (Fig. 6G, Supplementary Table 6), with some of them being driven by SEs while others driven by typical enhancers (TEs) (Fig. 6H). Taken together, our results showed that CASZ1b cross-talked with CRC TFs, repressed cell cycle regulators, mesenchymal genes and upregulated neural differentiation genes through remodeling enhancer activity to induce NB cell differentiation (Fig. 7).

## Discussion

How genetic or epigenetic alterations subvert a normal lineage specifying core regulatory circuitry to cause cancer is an area of intense investigation. In NB, chromosomal translocations [48] and genetic polymorphisms generating neo transcription binding sites [49] have been implicated in the dysregulated activity of the CRC regulating neural crest progenitors leading to NB. In this study, we examined the molecular mechanisms by which restoration of the NB Chr1p36 tumor suppressor CASZ1 regulates gene transcription and whether CASZ1 cross-talks with the NB CRC. We find that *CASZ1* is repressed by CRC components of noradrenergic NB. The restoration of CASZ1b directly represses CRC components, mesenchyme development genes, cell cycle regulators and upregulates neuronal genes by remodeling TEs and SEs to switch NB cells from a malignant noradrenergic phenotype to a more differentiated noradrenergic phenotype.

We find that CASZ1 cross-talks with critical neural crest lineage regulators and noradrenergic NB CRC components such as HAND2, GATA3 and TBX2 to suppress NB growth and enhance noradrenergic neuronal differentiation. This role is reminiscent of the role that CASZ1 plays in drosophila neuroblasts as well as mouse retina and myoblasts. The drosophila homolog of *CASZ1* cross-talks with other TFs to determine the ability of neuroblasts to generate progeny with distinct differentiation states [18–22]. For example, Hunchback activates *Kruppel*, Kruppel activates *Pdm* (*Pdm* 1 and 2), Pdm activates *CASZ1*, while Kruppel represses *CASZ1* and CASZ1 represses *Pdm* expression, these regulatory interactions ensure the sequential progression of temporal states during lineage development [18]. Aspects of this TF logic have also been demonstrated in mouse retinal cell development [15]. In myoblasts, *CASZ1* expression is activated by MYOD and MYOG, and CASZ1 activates *MYOD* and *MYOG*, while repressing *MYF5* to regulate and induce skeletal myogenic differentiation [33]. The common thread from these studies and our study indicates that CASZ1 regulates cell-fate decisions in different tissue types by cooperating with lineage-specific TFs.

A CRC is comprised of a small set of core TFs that form an interconnected, autoregulatory feed-forward loop that dominates and controls the expression of specific gene programs to maintain a specific cell type. Recent bioinformatic analyses generated CRC models for 75 human cell and tissue types [13]. The core TFs of a CRC are generally expressed in a lineage-specific manner and can reprogram cells from one type to another. Thus, the expression of these TFs needs to be precisely regulated during lineage specification. For example, during cell differentiation, TFs required for a differentiation state need to be upregulated, while TFs that are highly expressed to maintain a stem cell-like status need to be silenced. During normal peripheral nervous system development, a set of TFs including PHOX2B, HAND2, PHOX2A, HAND1, GATA2, GATA3 and ASCL1 interacts as a network to determine sympathetic neuronal differentiation [1, 37, 42, 43, 50, 51]. In NB, the neural crest lineage specifiers PHOX2B, HAND2, GATA3, ISL1, and TBX2 form a CRC to determine a malignant noradrenergic phenotype [10]. Recent studies showed that ISL1 regulates multiple oncogenic genes that are essential for the proliferation and differentiation of NB and sympathetic neurons [39, 52]. In this study, we found that the CRC components HAND2 and TBX2 repress *CASZ1* expression but upon increasing the levels of CASZ1 there is direct repression of the expression of the CRC components *PHOX2B, GATA3, ISL1* and *TBX2* in NB cells. This provides a novel model in which the CRC of cell type A directly represses a cell type B lineage-specific TF. The forced expression of this B lineage TF in cell type A will repress the cell type A CRC and induce a cell type B phenotypic switching. This formation of an interconnected feedback loop between a cell type specific TF and the cell type specific CRC might be a general rule that determines the specification of a cell’s fate and its commitment.

The results of single-cell studies on normal neural crest development of the sympathoadrenal system and our results support an important role for CASZ1 in neural crest lineage differentiation. It is beyond the scope of this study to characterize the role of CASZ1 in a normal neural crest development model, but our results provide interesting insights into how loss of a tumor suppressor gene such as CASZ1 might contribute to the oncogenic dysregulation of lineage specifying CRC such as occurs in NB. Our study provides support that the loss of a key inducer or amplifier of differentiation such as CASZ1 could hamper a cell’s ability to undergo terminal differentiation, which results in increased cellular plasticity.

Our study also provides mechanisms by which CASZ1 suppresses NB growth and induces noradrenergic neuronal differentiation. We have previously shown that the restoration of CASZ1 leads to an increase in the percentage of cells in the G1 phase of the cell cycle and delays the cell cycle progression [25]. In this study, we find that CASZ1 directly represses cell cycle regulators such as *CDK1, CDK6* and *CCND1*, which is accompanied by a decrease of H3K27ac signal and decreased transcription levels (Fig. 6F, Supplementary Fig. 3F). Dysregulation of cycle regulators such as *CCND1* and *CDK6* are implicated in NB, which make them therapeutic targets in NB [47, 53]. This direct repression of cell cycle regulators by CASZ1 identifies a mechanism by which *CASZ1* suppresses NB growth. The restriction in proliferative capacity and exit of NB cells from the cell-cycle after *CASZ1* restoration provides opportunities for post-mitotic cellular differentiation. Indeed, the restoration of *CASZ1* upregulates neuronal differentiation genes expressed in sympathoadrenal cells (Fig. 2A). Importantly, the increased expression results in CASZ1 direct binding to the enhancers of the neuronal genes and is accompanied by an increase signals H3K27ac signal at these genomic loci (Fig. 6). These results indicate that CASZ1 down-regulates cycle regulators and upregulates neuronal genes through remodeling enhancer activity.

In summary, our results indicate that the regulation of NB differentiation programs by CASZ1 is integral to a specific TF network. The NB CRC maintains a malignant noradrenergic phenotype, in part due to the loss of *CASZ1* dependent negative feedback regulatory circuit. The loss of the *CASZ1* tumor suppressor in NB tumors occurs through Chr1p LOH and/or PRC2 mediated epigenetic suppression [29]. Restoration of *CASZ1* re-establishes the negative feedback regulatory circuit with this established CRC, directly suppresses *CDKs* and *cyclins*, suppresses mesenchymal genes and activates neuronal differentiation genes to switch the malignant NB noradrenergic phenotype to a more differentiated noradrenergic phenotype. Our discovery suggests a novel model in which the CRC forms an interconnected negative feedback loop with a TF to control cell identity during cell-fate specification.

## Materials and methods

### Cell culture

Neuroblastoma cell lines SH-SY5Y (SY5Y), SK-N-AS (AS), and IMR32 were obtained from the cell line bank of the Pediatric Oncology Branch of the National Cancer Institute and have been genetically verified. All the NB cells were maintained in RPMI1640 media supplemented with 10% fetal calf serum (FBS) as well as 100 µg/mL streptomycin, 100 U/mL penicillin, and L-glutamine. Cells are grown at 37 ^o^C with 5% CO_2_. SY5YtetCASZ1b cells or AStetCASZ1b cells were generated by first stably transfected cell lines with pcDNA6TR vector followed by stable transfected with pT-Rex-DEST30 containing N-terminal FLAG tagged CASZ1b. SY5YtetCASZ1b and AStetCASZ1b cell lines are single clone selected and are cultured in the complete RPMI containing 5 µg/ml blasticidin and 500 µg/ml geneticin. CASZ1b expression is tetracycline (Tet) (1 µg/ml) inducible. Control or CASZ1 stable knockdown SY5Y cell lines are generated by infecting SY5Y cells with either control shRNA lentiviral particles (SigmaAldrich, MISSION pLKO.1-puro. Catalog SHC002V) or CASZ1 shRNA lentiviral particles (SigmaAldrich, shCASZ1_2, catalog TRCN0000129821; shCASZ1_3, catalog TRCN0000131216) followed by puromycin selection. Cell confluency assays using Essen IncuCyte ZOOM or FLR evaluate relative cell number in realtime. Cell neurite length was measured using Essen IncuCyte ZOOM neurite analysis software.

### Realtime PCR

Total mRNA was collected using the RNeasy Plus Mini Kit (Qiagen) as per the manufacturer’s protocol. Quantitative measurements of total β-actin and other genes’ levels were obtained using the BIO-RAD CFX Touch Realtime (RT) PCR detection system and performed in triplicate. Ct values were standardized to β-actin levels. Data from biological triplicates were shown in this study if not specifically mentioned in figure legend. Primer sequences used for realtime PCR are shown below:

**Table:** 

Gene	Forward Primer	Reverse Primer
Beta-Actin	GCCAACCGCGAGAAGATGA	CATCACGATGCCAGTGGTA
CASZ1	CAAAACAGACTCCATCACCACG	GTGCTGGCTGCCCGAGAAC
HTR3A	GCTGCGTCACCTGGTTCTGG	TGTCCCTCGGGCTCTTCTCG
NGFR	ACCTCATCCCTGTCTATTGCTCC	GCTGTTGGCTCCTTGCTTGTT
TH	CCTACCAAGACCAGACGTACCAGTCA	TGCACCTAGCCAATGGCACTCA
GATA3	ACCACAACCACACTCTGGAGGA	TCGGTTTCTGGTCTGGATGCCT
HAND2	GGCAGAGATCAAGAAGACCGAC	CGGCCTTTGGTTTTCTTGTCGTT
ISL1	GGCATGTTTGAAATGTGCGG	ACACAGCGGAAACACTCGAT
PHOX2B	CCTGAAGATCGACCTCACAGAG	TTTTGCCCGAGGAGCCGTTCTT
TBX2	GGCCTTCCACAAGCTGAAG	GCGGCTGGTACTTGTGCAT

### Antibodies

The antibodies used for western blot, immunofluorescence staining and ChIP-seq are obtained from different companies. Antibodies from ThermoFisher: GAP43 (Cat. # PA1-16729, 1:250 for immunofluorescence), goat anti-mouse IgG Alexa Fluor 594 (Cat. # A-11032, 1:250 for immunofluorescence staining). Antibodies from SigmaAldrich: FLAG (Cat. # F1804, 1:1000 for western blot) Antibodies from Santa Cruz: GAPDH (Cat. # sc-25778, 1:2000 for western blot), goat anti-mouse IgG HRP secondary antibody (Cat. # sc-2005, 1:2000), goat anti-rabbit IgG HRP secondary antibody (Cat. # sc-2004, 1:2000). Antibody from Abcam: H3K27ac (Cat. # ab4729, 4 µg/reaction for ChIP), HAND2 (Cat. # ab200040, 4 µg/reaction for ChIP). Antibody from Active Motif: RNA polymerase II (Cat. # 39097, 4 µg/reaction for ChIP). Antibody generated by collaborating with Rockland Immunochemicals Inc: CASZ1 (1:4000 for western blot, 4 µg/reaction for ChIP). Antibody from EMD Millipore: H3K27me3 (Cat. # 07-449, 4 µg/reaction for ChIP). Antibody from Cell Signaling Technology, H3K4me3 (Cat. # 9751, 4 µg/reaction for ChIP).

### Protein isolation and western blot analysis

For assessment of protein levels, cells were lysed using RIPA buffer, and 10 µg of total protein was separated and electroblotted as described previously [28]. Protein bands were detected using a goat anti-rabbit or mouse IgG-HRP conjugated secondary antibody (200 µg/mL; Santa Cruz Biotechnology) and visualized using enhanced chemiluminescence (Amersham Biosciences).

### Immunofluorescence

Cells were cultured in 8-well Lab-Tek Chamber Slides (Cat. No. 177402) for indicated time. Cells were fixed, permeabilized, blocked, and stained as described previously [28]. For indirect immunofluorescent cell staining, an anti-GAP43 monoclonal antibody and an Alexa Fluor 594-conjugated goat anti-mouse antibody were used to detect GAP43. Stained cells were imaged and analyzed using a Nikon Eclipse TE300 fluorescent microscope.

### RNA-seq

Total RNA was isolated and subjected to RNA-seq analysis from SY5YetCASZ1b cells treated with or without Tet for 48 h, and AStetCASZ1b cells that were treated with or without Tet for 72 h. Total RNA extraction was carried out using a RNeasy Plus Mini Kit (Qiagen Inc.) according to the manufacturer’s instructions. Strand-specific whole transcriptome sequencing libraries were prepared using TruSeq® Stranded Total RNA LT Library Prep Kit (Illumina, San Diego, CA, USA) by following the manufacturer’s procedure. RNA-seq libraries were sequenced on Illumina HiSeq 4000 of paired-end with read length of 150 bp, or Novaseq S1 of paired-end with read length of 100 bp. The Fastq files with 150 bp or 100 bp paired-end reads were processed using Partek Flow or R package Deseq2. In brief, the raw reads are aligned using STAR and the aligned reads are quantified to annotation model through Partek E/M. The normalization method used here is counts per million (CPM) through Partek Flow. The normalized counts were then subjected to statistic analysis using GSA or ANOVA. To get T-scores, the normalized counts acquired from Partek Flow are exported and further analyzed using Parteck Genomics Suite v7.17. To eliminate batch effect, some of the CPM from Partek Flow were analyzed using DESeq2. Statistical results of differentially expressed genes from Partek Flow, or Parteck Genomics Suite v7.17 or DESeq2 were analyzed using QIAGEN’s Ingenuity® Pathway Analysis (IPA®, QIAGEN) and gene set enrichment analysis (GSEA) (http://www.broadinstitute.org/gsea/index.jsp). By default, the false discovery rate (FDR) less than 0.25 is considered significant in GSEA.

### ChIP-seq

ChIP was performed using the ChIP-IT High Sensitivity kit (Active Motif) as per the manufacturer’s instruction. Briefly, formaldehyde (1%, 13–15 min) fixed cells were sheared to achieve chromatin fragmented to a range of 200–700 bp using an Active Motif EpiShear Probe Sonicator. SY5YtetCASZ1b cells were sonicated at 30% amplitude, pulse for 20 s on and 30 s off for a total sonication “on” time of 15 min. Sheared chromatin samples were immunoprecipitated overnight at 4 °C with antibodies targeting CASZ1, H3K27ac and RNA Pol II. IMR32 cells were sonicated at 25% amplitude, pulse for 20 s on and 30 s off for a total sonication “on” time of 16 min. Sheared chromatin samples were immunoprecipitated overnight at 4 °C with antibodies targeting HAND2, H3K27ac, H3K4me3, and H3K27me3. ChIP-seq DNA libraries were prepared by Frederick National Laboratory for Cancer Research sequencing facility. Libraries were multiplexed and sequenced using TruSeq ChIP Samples Prep Kit (75 cycles), cat. # IP-2-2-1012/1024 on an Illumina NextSeq machine. 25,000,000–30,000,000 unique reads were generated per sample. All the home generated ChIP-seq datasets can be found in the Gene Expression Omnibus (GEO) database.

### ChIP-seq data processing

ChIP-enriched DNA reads were mapped to reference human genome (version hg19) using BWA [54]. Duplicate reads were infrequent but discarded. For IGV sample track visualization, coverage density maps (tdf files) were generated by extending reads to the average size (measured by Agilent Bioanalyzer minus 121 bp for sequencing adapters) and counting the number of reads mapped to each 25 bp window using igvtools (https://www.broadinstitute.org/igv/igvtools).

ChIP-seq read density values were normalized per million mapped reads. High-confidence ChIP-seq peaks were called by MACS2 (https://github.com/taoliu/MACS) with the narrow peak calling. The peaks which overlapped with the possible anomalous artifact regions (such as high-mappability regions or satellite repeats) blacklisted by the ENCODE consortium (https://sites.google.com/site/anshulkundaje/projects/blacklists) were removed using BEDTools. Peaks from ChIP-seq of CASZ1, H3K27ac and RNA Pol II in SY5YtetCASZ1b cells were selected at a stringent *p*-value (~*p* < 10^−7^). Peaks within 1,000 bp to the nearest TSS were set as promoter. The distribution of peaks (as intronic, intergenic, exonic, etc.) was annotated using HOMER. Enrichment of known and de novo motifs were found using HOMER script “findMotifsGenome.pl” (http://homer.salk.edu/homer/ngs/peakMotifs.html). Heatmaps of signal intensity of ChIP samples were generated using deepTools. Briefly, computeMatrix was used to calculate signal intensity scores per ChIP sample in a given genome region that was specified by a bed file. The output of computeMatrix was a matrix file of scores of two ChIP samples which was then used to generate the heatmap using plotHeatmap. Metagene plots of ChIP-seq data were also performed using ComputeMatrix function of the deepTools [55].

The enhancers were identified using the ROSE2 (Rank Order of Super-Enhancers) software (https://github.com/BradnerLab/pipeline), using distal (>2500 bp from TSS) H3K27ac peaks [56, 57]. Enhancer constituents were stitched together if clustered within a distance of 12.5 kb. The enhancers were classified into typical and super-enhancers based on a cutoff at the inflection point in the rank ordered set (where tangent slope = 1) of the ChIP-seq signal (input normalized) with *p* < 10^−7^.

Among all the CASZ1b binding sites, promoter regions were defined directly from the *Homer* annotated bed file within 1000 bp of the TSS. The remaining CASZ1 binding sites in the non-promoter regions were divided into active enhancer-associated regions if they overlap with the enhancers (defined by H3K27ac ChIP-seq) and others if they do not overlap with enhancers.

To determine the alterations on enhancers when CASZ1 is restored in SMS-CTR cells, signal intensity of H3K27ac ChIPseq+/−3K of CASZ1b peak centers was extracted and compared between control and Tet treated cells. Briefly, bed files of all H3K27ac that overlapped with CASZ1b peaks were extracted. ComputeMatrix function of the deepTools was used to generate a matrix of signal intensity of H3K27ac up- and down-stream of the CASZ1b peak centers, as intensity scores in 10 bp bins.

### Statistics and reproducibility

The statistical analyses used throughout this paper are specified in the appropriate results paragraphs and Methods sections. Additional statistical analyses were performed using Microsoft Excel, standard two-tailed Student’s *t* test, one-way ANOVA and the software GraphPad Prism 8.1.0. A representative experiment such as micrographs has been repeated at least two to three times.

## Supplementary information

Table S1

Table S2

Table S3

Table S4

Table S5

Table S6

Supplementary figures

Reproducibility checklist

## Data Availability

All the home generated RNA-seq and ChIP-seq datasets can be found in the Gene Expression Omnibus (GEO) database. GEO accession number for data generated in this study is GSE182871 (ChIP-seq in SY5Y cells), GSE182872 (RNA-seq in AS cells) and GSE182873 (RNA-seq in SY5Y cells). GEO accession number for HAND2 ChIP-seq experiment done in IMR32 cells is GSE184058 [40]. GEO accession numbers or SRA accession numbers for publicly available ChIP-seq data are: GSE94822 for ChIP-seq experiments done in BE2C; SRX4623679, GSE65664, GSE80197 for ChIP-seq experiments done in SY5Y cells. To evaluate CASZ1 and CRC components mRNA levels in adrenal gland, NB cell lines and patients, we queried microarray data deposited in R2 database (https://hgserver1.amc.nl/cgi-bin/r2/main.cgi).
