# Supplementary material for: Loss of CASZ1 tumor suppressor linked to oncogenic subversion of neuroblastoma core regulatory circuitry
Source: Cell Death Dis. 2022 Oct 15;13(10):871. doi: 10.1038/s41419-022-05314-6 (PMC9569368; doi:10.1038/s41419-022-05314-6)
Supplement: Supplementary file 7 — Supplementary figures [file 41419_2022_5314_MOESM7_ESM.docx]

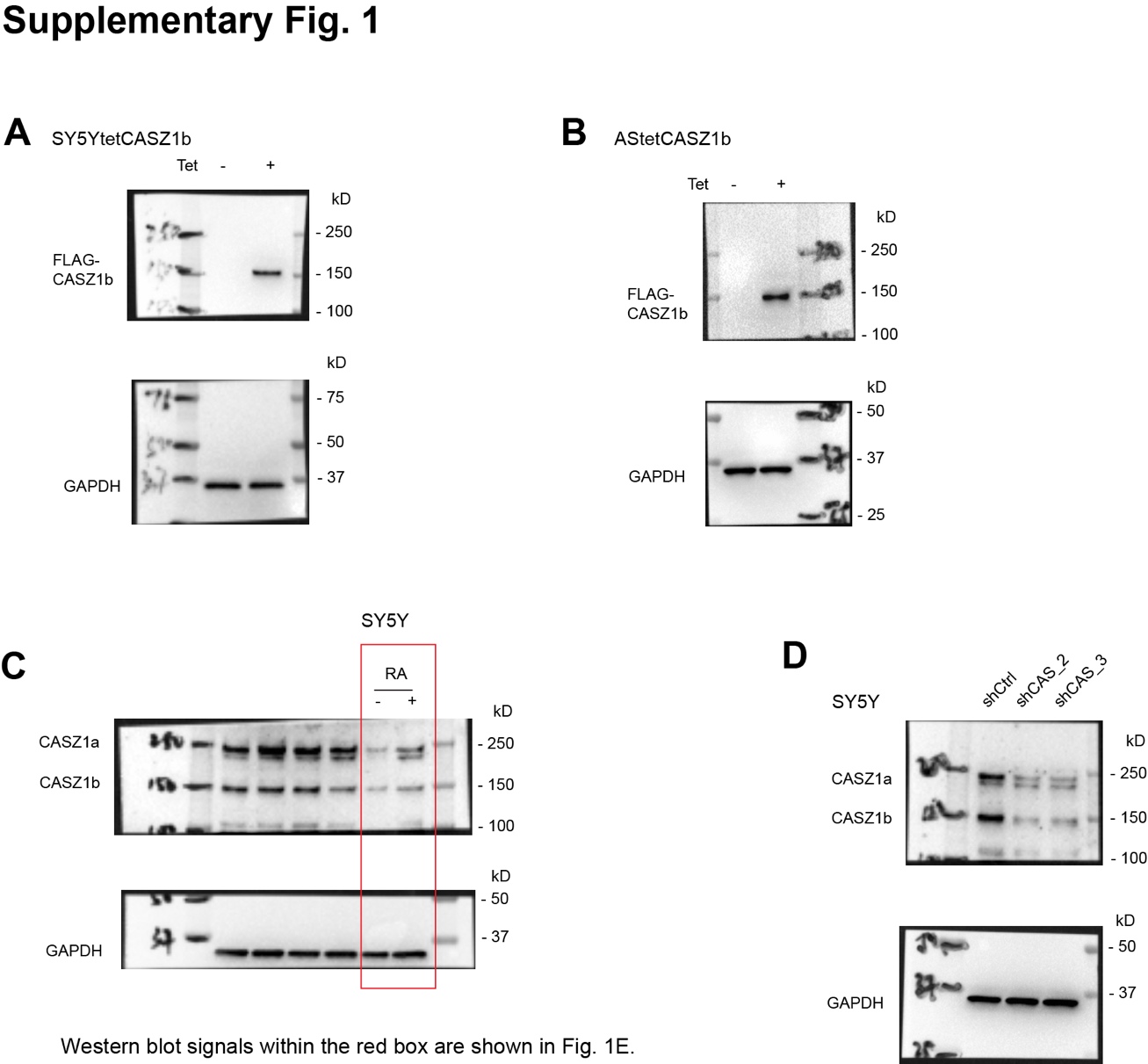


**Supplementary Fig. 1. Uncropped immunoblots of the different figures.** **A**, Immunoblots related to Fig. 1A. **B**, Immunoblots related to Fig. 1B. **C**, Immunoblots related to Fig. 1E. Western blots within the red box are shown in Fig. 1E. **D**, Immunoblots related to Fig. 1F.


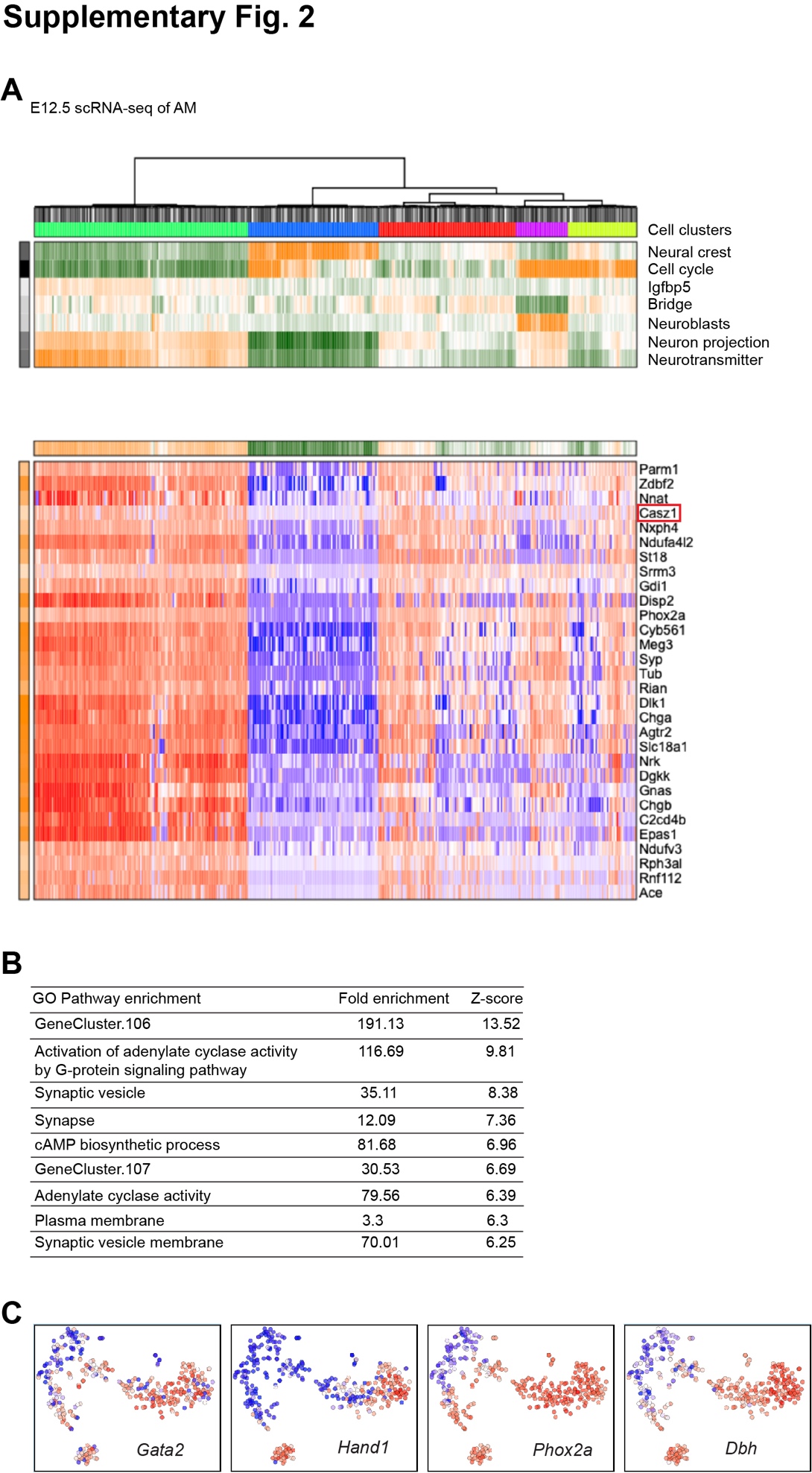


**Supplementary Fig. 2. The expression pattern of *Casz1* in E12.5 mouse embryonic adrenal medulla at single cell level**. **A,** Cell are clustered based on gene expression (top panel). The cells enriched in genes co-expressed with *Casz1* (bottom panel) including *Phox2a*, *Chga* and *Chgb* are similar to clusters of cells that are enriched in neuron projection and neurotransmitter genes (top panel). Expression magnitude: green (or blue) low, white intermediate and red high. Harvard interactive interface tools was used to generate the heatmap (http://pklab.med.harvard.edu/cgi-bin/R/rook/nc.SS2_16_250-2/index.html; scRNAseq results are available at Gene Expression Omnibus, GSE99933). **B,** GO pathway enrichment assay of genes co-expressed with Casz1 shows a positive enrichment of genes involved in neuron projection. **c,** The single cell mRNA expression pattern of sympathoadrenal genes in E12.5 mouse embryonic adrenal medulla were analyzed using the Harvard interactive interface tools (<http://pklab.med.harvard.edu/cgi-bin/R/rook/nc.SS2_16_250-2/index.html>).


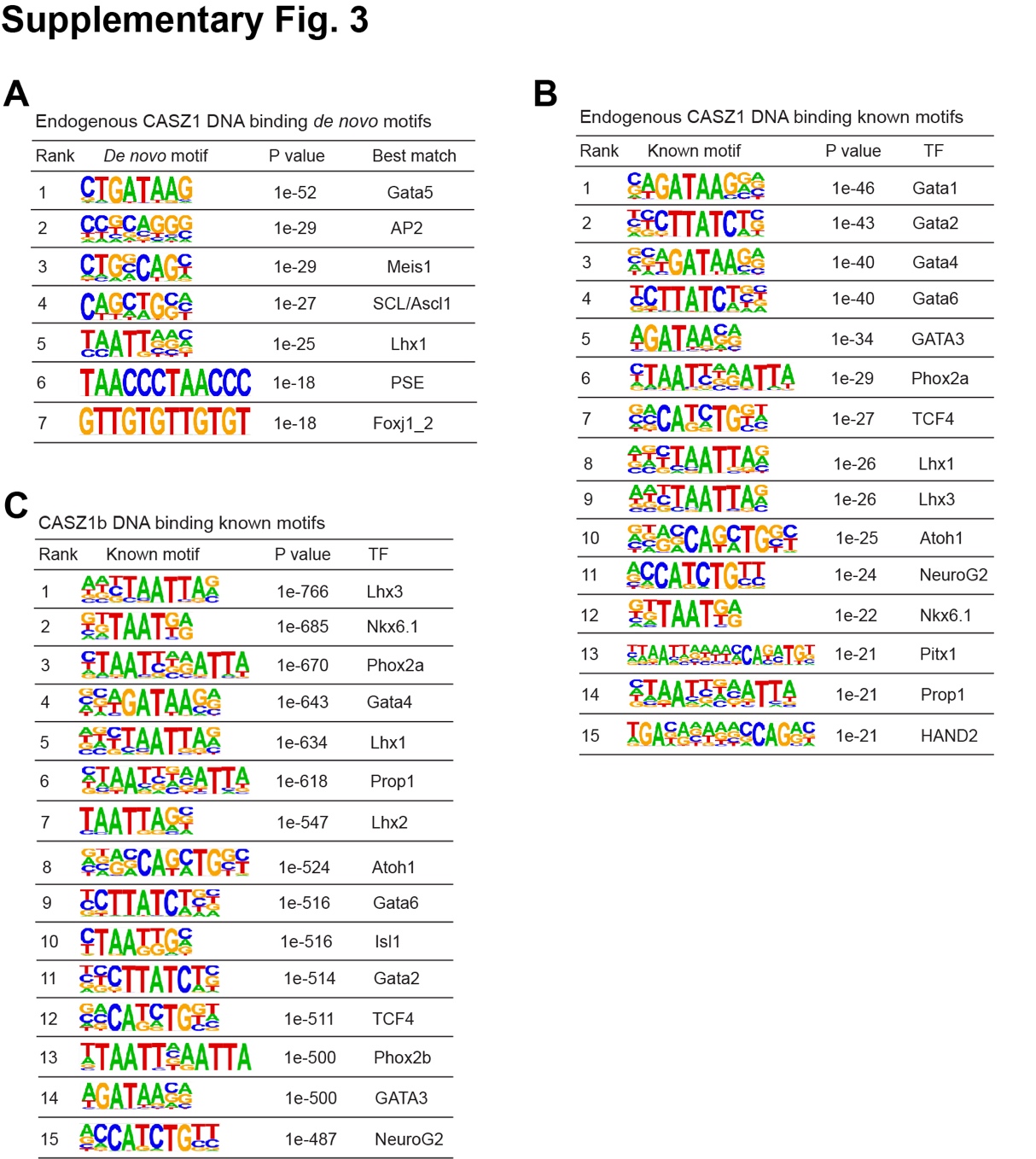


**Supplementary Fig. 3. CASZ1 binding motifs**. **A,** Homer *de novo* motif scan of the endogenous CASZ1 binding sites. **B,** Homer known motif scan of the endogenous CASZ1 binding sites. **C,** Homer known motif scan of the restored CASZ1b binding sites.


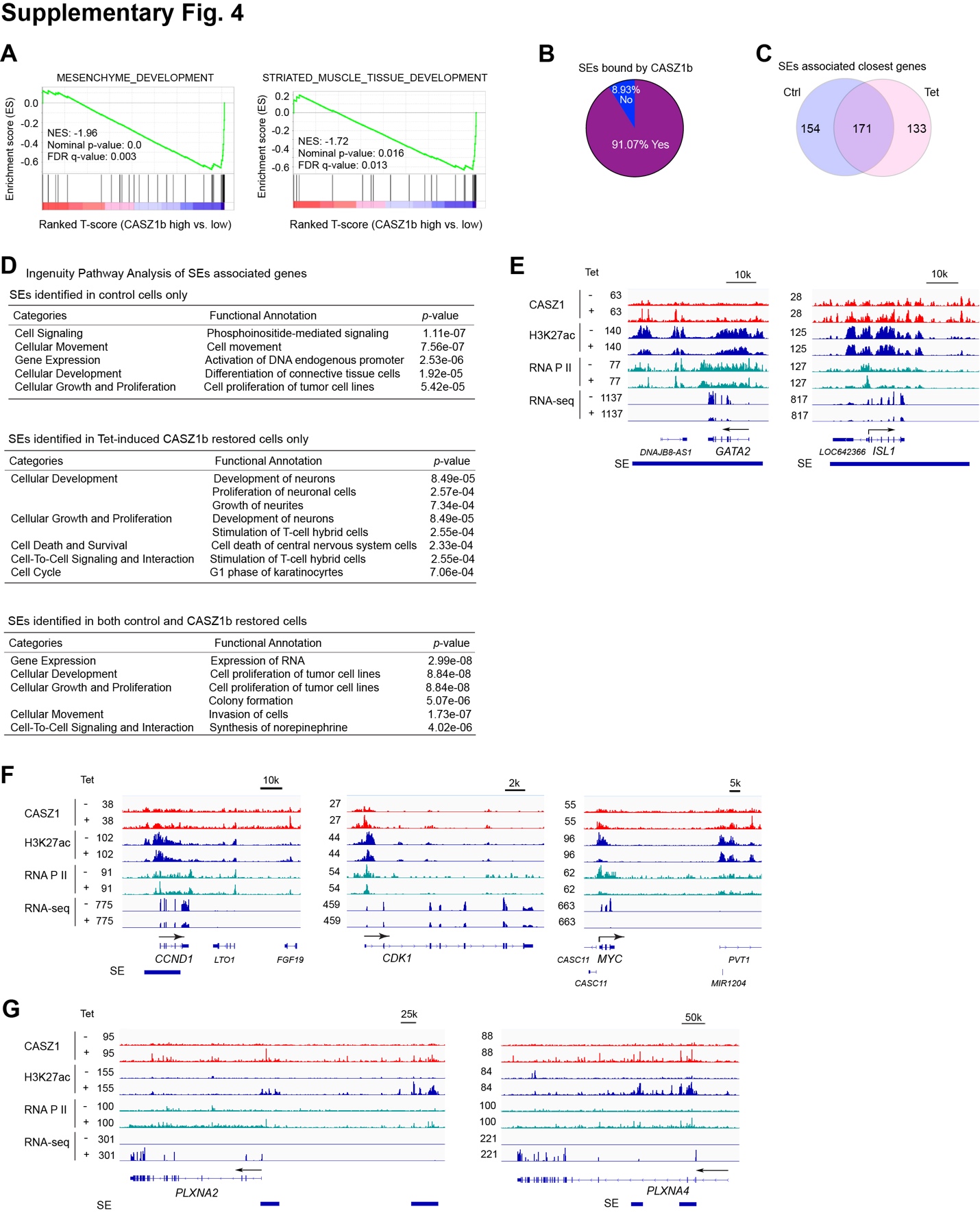


**Supplementary Fig. 4. CASZ1b regulates gene transcription through affecting enhancer activity. A,** GSEA shows the negative enrichment of mesenchyme development genes and striated muscle genes when CASZ1b is restored in SY5Y cells. **B,** Pie chart shows that CASZ1b binds to over 90% of SE when restored in SY5Y cells. **C,** Venn diagram shows the gain and loss of SEs before and after CASZ1 is restored in SY5Y cells. Here only focused on the closest genes associated SEs. **D,** Ingenuity Pathway Analysis indicates that the lost SEs associated genes after the restoration of CASZ1b are enriched in regulating “cell proliferation of tumor cell lines” (top panel**)**, but the gained SEs associated genes are enriched in “development of neurons” and “growth of neurites” (middle panel), while the common SEs associated genes are enriched in “cell proliferation of tumor cell lines” and “synthesis of norepinephrine” (bottom panel). **E,** Signal tracks show that the restoration of CASZ1b in SY5Y cells results in a decrease of SE signal (H3K27ac), as well as RNA Pol II signal and RNA-seq reads on *GATA2*, *ISL1 and TBX2.* **F,** Signal tracks show that the restoration of CASZ1b in SY5Y cells results in a decrease of SE signal or TE signal (H3K27ac), as well as RNA Pol II signal and RNA-seq reads on *CCND1*, *CDK1 and MYC.* **G,** The restoration of CASZ1b in SY5Y cells results in an increase of SE signal (H3K27ac), as well as RNA Pol II signal and RNA-seq reads on the neuronal gene *PLXNA2* and *PLXNA2*.
