# Supplementary material for: Loss of CASZ1 tumor suppressor linked to oncogenic subversion of neuroblastoma core regulatory circuitry
Source: Cell Death Dis. 2022 Oct 15;13(10):871. doi: 10.1038/s41419-022-05314-6 (PMC9569368; doi:10.1038/s41419-022-05314-6)
Supplement: Supplementary file 8 — Reproducibility checklist [file 41419_2022_5314_MOESM8_ESM.pdf]

Corresponding Author Name: Zhihui Liu
Manuscript Number:CDDIS-22-2808-T

SPRINGER NATURE

Reporting Summary

Springer Nature wishes to improve the reproducibility of the work that we publish. This checklist is used to ensure good reporting standards and to improve the reproducibility. Please respond completely to all questions relevant to your manuscript. For more information, please read the journal's Guide to Authors.

☑ **Check here to confirm that the following information is available in the Material & Methods section:**

- **The exact sample size (*n*)** for each experimental group/condition, given as a number, not a range
- **A description of the sample collection** allowing the reader to understand whether the samples represent technical or biological replicates (including how many animals, litters, culture, etc.)
- **A statement of how many times the experiment shown was replicated in the laboratory**
- **Definitions of statistical methods and measures**: For small sample sizes (n<5) descriptive statistics are not appropriate, instead plot individual data points
  - Very common tests, such as *t*-test, simple χ^2 tests, Wilcoxon and Mann-Whitney tests, can be unambiguously identified by name only, but more complex techniques should be described in the methods section
  - Are tests one-sided or two-sided?
  - Are there adjustments for multiple comparisons?
  - **Statistical test results**, e.g., ***P* values**
  - Definition of **'center values'** as **median or mean**;
  - Definition of **error bars as s.d. or s.e.m. or c.i.**

Please ensure that the answers to the following questions are reported in the manuscript itself. We encourage you to include a specific subsection in the methods section for statistics, reagents and animal models. Below, provide the page number or section and paragraph number.

Statistics and general methods

	**Reported in section/paragraph or page #**
1. How was the sample size chosen to ensure adequate power to detect a pre-specified effect size? (Give section/paragraph or page #)	Sample size was chosen to give enough statistical power.
For animal studies, include a statement about sample size estimate even if no statistical methods were used.	Not applicable.
2. Describe inclusion/exclusion criteria if samples or animals were excluded from the analysis. Were the criteria pre-established? (Give section/paragraph or page #)	No data were excluded.
3. If a method of randomization was used to determine how samples/animals were allocated to experimental groups and processed, describe it. (Give section/paragraph or page #)	Not applicable.
For animal studies, include a statement about randomization even if no randomization was used.	Not applicable.

4. If the investigator was blinded to the group allocation during the experiment and/or when assessing the outcome, state the extent of blinding. (Give section/paragraph or page #)	The investigators were not blinded to group allocation during experiments. Conclusions were made based on quantitative parameters and statistical significance of the data.
For animal studies, include a statement about blinding even if no blinding was done.	Not applicable.
5. For every figure, are statistical tests justified as appropriate?	The statistical test used in each figure is indicated in the figure legends.
Do the data meet the assumptions of the tests (e.g., normal distribution)?	Given that sample size is less than 10, we assume that the data did not follow Gaussian distribution and therefore [illegible]
Is there an estimate of variation within each group of data?	No estimation of variation within each group was compute.
Is the variance similar between the groups that are being statistically compared? (Give section/paragraph or page #)	We used non parametric test to compare the means within the groups, it does not require estimation of the variance.

Reagents

	**Reported in section/paragraph or page #**
6. Report the source of antibodies (vendor and catalog number)	The source of antibodies is reported in Materials and Methods section.
7. Identify the source of cell lines and report if they were recently authenticated (e.g., by STR profiling) and tested for mycoplasma contamination	The source of cell lines is reported in Materials and Methods section and they were authenticated, tested free for mycoplasma contamination.

Animal Models

	**Reported in section/paragraph or page #**
8. Report species, strain, sex and age of animals	Not applicable.
9. For experiments involving live vertebrates, include a statement of compliance with ethical regulations and identify the committee(s) approving the experiments.	Not applicable.

10. We recommend consulting the ARRIVE guidelines (*PLoS Biol.* **8**(6), e1000412,2010) to ensure that other relevant aspects of animal studies are adequately reported.

Human subjects

Reported in section/paragraph or page #

Item	Reported in section/paragraph or page #
11. Identify the committee(s) approving the study protocol.	Not applicable.
12. Include a statement confirming that informed consent was obtained from all subjects.	Not applicable.
13. For publication of patient photos, include a statement confirming that consent to publish was obtained.	Not applicable.
14. Report the clinical trial registration number (at ClinicalTrials.gov or equivalent).	Not applicable.

15. For phase II and III randomized controlled trials, please refer to the CONSORT statement and submit the CONSORT checklist with your submission.

16. For tumor marker prognostic studies, we recommend that you follow the REMARK reporting guidelines.

Data deposition

Reported in section/paragraph or page #

Item	Reported in section/paragraph or page #
17. Provide accession codes for deposited data. Data deposition in a public repository is mandatory for:<br>a. Protein, DNA and RNA sequences<br>b. Macromolecular structures<br>c. Crystallographic data for small molecules<br>d. Microarray data	All the home generated RNA-seq and ChIP-seq datasets can be found in the Gene Expression Omnibus (GEO) database. GEO accession number for data generated in this study is GSE182871 (ChIP-seq in SY5Y cells), GSE182872 (RNA-seq in AS cells) and GSE182873 (RNA-seq in SY5Y cells).

Deposition is strongly recommended for many other datasets for which structured public repositories exist; more details on our data policy are available in the Guide to Authors. We encourage the provision of other source data in supplementary information or in unstructured repositories such as Figshare and Dryad. We encourage publication of Data Descriptors (see Scientific Data) to maximize data reuse.

Item	Reported in section/paragraph or page #
18. If computer code was used to generate results that are central to the paper's conclusions, include a statement in the Methods section under "**Code availability**" to indicate whether and how the code can be accessed. Include version information as necessary and any restrictions on availability.	Not applicable.
